# Effect of Methodological and Ecological Approaches on Heterogeneity of Nest-Site Selection of a Long-Lived Vulture

**DOI:** 10.1371/journal.pone.0033469

**Published:** 2012-03-08

**Authors:** Rubén Moreno-Opo, Mariana Fernández-Olalla, Antoni Margalida, Ángel Arredondo, Francisco Guil

**Affiliations:** 1 CBD-Habitat Foundation, Madrid, Spain; 2 Technical School of Forestry, Universidad Politécnica de Madrid, Madrid. Spain; 3 Bearded Vulture Study and Protection Group, El Pont de Suert, Lleida, Spain; 4 Division of Conservation Biology, Institute of Ecology and Evolution, University of Bern, Bern, Switzerland; University of Western Ontario, Canada

## Abstract

The application of scientific-based conservation measures requires that sampling methodologies in studies modelling similar ecological aspects produce comparable results making easier their interpretation. We aimed to show how the choice of different methodological and ecological approaches can affect conclusions in nest-site selection studies along different Palearctic meta-populations of an indicator species. First, a multivariate analysis of the variables affecting nest-site selection in a breeding colony of cinereous vulture (*Aegypius monachus*) in central Spain was performed. Then, a meta-analysis was applied to establish how methodological and habitat-type factors determine differences and similarities in the results obtained by previous studies that have modelled the forest breeding habitat of the species. Our results revealed patterns in nesting-habitat modelling by the cinereous vulture throughout its whole range: steep and south-facing slopes, great cover of large trees and distance to human activities were generally selected. The ratio and situation of the studied plots (nests/random), the use of plots vs. polygons as sampling units and the number of years of data set determined the variability explained by the model. Moreover, a greater size of the breeding colony implied that ecological and geomorphological variables at landscape level were more influential. Additionally, human activities affected in greater proportion to colonies situated in Mediterranean forests. For the first time, a meta-analysis regarding the factors determining nest-site selection heterogeneity for a single species at broad scale was achieved. It is essential to homogenize and coordinate experimental design in modelling the selection of species' ecological requirements in order to avoid that differences in results among studies would be due to methodological heterogeneity. This would optimize best conservation and management practices for habitats and species in a global context.

## Introduction

To date numerous studies have evaluated the relationships between one or various threatened species and the environmental variables at work in the habitats in which they carry out the distinct phases of their life cycles [1]. Of these, one of the commonest areas of study is research into the factors affecting reproductive processes, which have serious repercussions for population dynamics, and which have become one of the most important lines of work in conservation biology [2], [3]. In general, the extent of our knowledge of the factors that determine reproduction depends on natural processes and/or human activities, but it is also influenced by the methodology employed in research [4], [5]. Thus, general conclusions regarding the ecological aspects affecting the choice of reproduction sites of a single species at different spatial scales and geographical locations has only been possible in a very few cases. A possible solution is the application of a meta-analysis in order to combine results of previous studies and draw general conclusions concerning the ecological and human factors that affect habitats and species under study [6]. In order to do so, this type of analysis has to overcome the difficulties posed by the need to standardize heterogeneous information, the deficiencies in data collection in certain analyses, the lack of unifying criteria in data recording and variations in the ecological requirements of the species being modelled [7], [8].

In light of these considerations and taking as a case study the cinereous vulture (*Aegypius monachus*) we evaluated the factors that determine breeding habitat selection by a new field study in Spain. This species is a good model for evaluating the conservation status of the ecosystems in which it breeds given its role in trophic chains by completing the processing cycle and assimilation of biomass of dead animals [9], [10] and its sensitivity to alterations affecting the landscapes it inhabits, such as non-compatible forestry practices or human disturbances [11], [12].

From a descriptive perspective, precedent studies showed common patterns of nesting-habitat selection by cinereous vulture [13]–[19]: nests were located in forests situated on mountain slopes with large trees and high vegetation cover, far away from the human presence. Nevertheless, there are also differences across the studies in the variables that were statistically significant as well as divergences in the applied methods that could affect the final results. Therefore, we evaluated as hypothesis how the methodological procedures influenced the variability reflected in the results of different studies and, as consequence, the nesting-habitat selection of the cinereous vulture at a global scale, through meta-analysis [20]. Meta-analysis is a statistical procedure for combining data from multiple studies by applying objective formulas with the purpose of evaluating the identification and reasons of the common findings or the variation among the results of the compared studies [20].

The general objectives of this work were thus:

- to know the environmental factors determining the nesting-habitat selection of the cinereous vulture in a breeding colony of central Spain,
- to study the causes of variation in results regarding nest-site preferences from different published studies, together with the present field study, all integrated in a meta-analysis, and
- to evaluate which of the statistically significant factors highlighted in each study are the most relevant to nest-site selection in a Palearctic context and how they are related to the vulnerability and the ecological characteristics of each studied population, as a way of establishing the most appropriate management and conservation measures.

## Methods

### The study species

The cinereous vulture is classified as near threatened (7 200–10 000 pairs) [21] and breeds from the Iberian Peninsula as far as Eastern Asia. This vulture can be considered as an habitat indicator species due to its large foraging range [9], the specificity of its food requirements [22], [23] and its nest-site selection in large mature trees [13]. This species' habitat is located in areas with high conservation status that are important to many other species, some of which are also threatened [24]–[26].

### Study area

**Figure 1:**
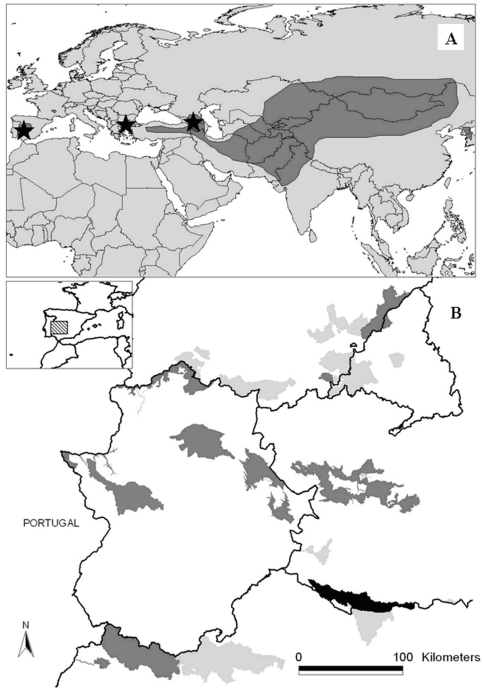
Global distribution range of cinereous vulture (dark grey) and metapopulations in which nest-site modelling studies and meta-analyses have been performed in this article (black stars). (A). Distribution in peninsular Spain of the Special Protected Areas (SPA) with presence of breeding cinereous vultures, specifying those in which nest-site modelling were studied (dark grey) and where the present field study was developed (black) (B).

The nest-site selection study was conducted in Alcudia and Sierra Madrona Natural Park, Spain (Figure 1), home to a colony of 129 pairs [27]. The site is part of an upland area (736–1 115 m a.s.l.) in which the dominant vegetation consists of typical Mediterranean trees such holm oak (*Quercus rotundifolia*), cork oak (*Quercus suber*), strawberry-tree (*Arbutus unedo*), prickly juniper (*Juniperus oxycedrus*) and Lusitanian oak (*Quercus faginea*), associated with a well-developed shrub layer.

### Field work and studied variables

In October-December 2005, once the breeding season was over, we visited all the cinereous vulture nests in the area (n = 155 nests). All nests, occupied in 2005 or unoccupied but with evidence of occupation in recent years, were studied [14], [28]. We also selected random points (n = 85) in areas within the perimeter of the breeding colony [15], [29]. We visited each point and recorded, from the nearest tree (since cinereous vulture breed in trees), the same data as for nests. The presence or absence of a nest at each point was established as a response variable [30].

The independent variables studied were chosen on the basis of previously evaluated aspects of this species' nest-site selection [15], [16] or as factors that relate to the land management [15]. Specifically, explanatory variables affecting factors relating to two spatial scales (microhabitat, n = 3, and landscape, n = 18) were selected.

As microhabitat variables we measured the tree characteristics in relation to the tree species (*Sp_tree*), the height (m) from a visual estimation of the tree where nest is present or the tree randomly selected (*H_tree*) and the diameter (cm) at breast height (dbh) of tree where nest is present or the tree randomly selected (*D_tree*).

For the landscape scale we considered 1) geomorpholocial variables as the altitude (m asl, *Alt*), the presence of nest/random tree in a natural scree (yes/no, *Scree*), the orientation of the slope where nest/random tree is located since all nests in the study area are situated in slopes greater than 15% (N, S, W, E, *Orient*), the slope of the hillside in a 100 m radius around the location of a nest/random tree (%, *Slope*) and the distance (m) from nest/random tree to nearest natural scree (*D_scree*), all of them calculated through GIS (ArcView 3.1 software) and aerial photographs; 2) vegetation variables, as the number of trees taller than 4 m existing in a 25 m radius around the nest/random tree through setting a survey plot and visual estimation of the height of the trees (*Rad25_tree*), the average high of the shrub in a 100 m radius around the nest/random tree through the measurement of the shrubs existing in four line-transects (*H_shrub*), the percentage of coverage in a 100 m radius around nest/random tree of trees (*%_tree*), shrub (%_*shrub*), pasturelands (*%_past*), scree or rock outcrop (*%_scree-rock*), cork oak tree (*%_Qsuber*), holm oak tree (*%_Qrot*) and other tree species (*%_othersp*) through a direct visual assessment in the field; and 3) human disturbance-related variables as the length (m) of unpaved tracks in a 500 m radius around the nest/random tree (*Long_tracks*) and the distances (m) from the nest/random tree to the nearest paved road (*D_road*), to the nearest building (*D_const*) and to the nearest unpaved track (*D_track*) through the application of geographic information systems (GIS, ArcView 3.1) to measure distances and length of tracks.

Climatic factors were not taken into account due to the relatively small study area (11 115 ha) and the homogeneity of vegetation structure and altitude intervals, which imply similar values of temperature, rainfall, humidity or solar radiation across the studied landscape.

### Data compilation from previous studies: meta-analysis

We carried out a bibliographical search of articles published on habitat selection in the cinereous vulture in peer-reviewed journals and official reports. We were able to collate data from seven articles referring to 15 colonies in three different metapopulations (Figure 1, see Appendix S1).

We generated two response variables as means of comparison of the following null-hypothesis: 1) absence of differences in the explained variability ( = variance or deviance) among the studies (Appendix S1). The variance and deviance were selected in order to compare the variability explained and, thus, the robustness of the results from all the analyzed studies [31], [32], but taking into account that deviance is related to the number of studied variables and the sample size (Appendix S1), and 2) no differences in the frequency of a variable resulting statistically significant in the analyzed studies. We assumed that a variable might better explain the general selection patterns if being significant more times. For this later variable we also considered a) its positive/negative (+/−) relationship with the presence of cinereous vultures, and b) the proportion of times that each variable was significant in relation to the number of times it was studied.

Additionally, explanatory variables related to the data-sampling methods (*Sampling methods*, n = 4) and to aspects regarding the vulnerability and ecology of the species (*Ecological-vulnerability*, n = 3) were identified from the different studies. *Sampling methods* variables were intended for a better detailing of the meta-analysis and thus were included into the assessment of the effect of the methodological procedures used in each study on the explained variability. The variables considered were the type of sample considered in the analysis (point or polygon, *Sample*), the assignment to data set in only one year (yes or no, *Year of sampling*), the proportion between the number of nest-samples and number of random samples (*Nest/random*) and the location of random plots (1 =  inside the perimeter of breeding colony; 2 =  forest habitat in and around breeding colony; 3 =  all habitats in and around the breeding colony, *Location of random samples*).

Studied breeding colonies were classified into *vulnerability* and *ecological* categories for a further analysis aiming at illustrating if any of the more significant kind of variables from those evaluated in the studies were related to the ecological and vulnerability characteristics of the studied populations. So, we identified as variables the vulnerability of the study area (1 =  less than 40 pairs in the breeding colony and less than 500 pairs at national level; 2 =  less than 40 pairs in the breeding colony and more than 500 pairs at national level; 3 =  more than 40 pairs in the breeding colony and more than 500 pairs at national level, *Threat level*), the number of breeding pairs in the colony (1≤30 pairs; 2>30 pairs and ≤100 pairs; 3>100 pairs, *Colony size*) and the type of vegetation (pine or oak, *Habitat*).

### Statistical analyses

#### Present field study

To study the factors that affect nest-site selection, we selected a total of 240 points, that were analyzed at both microhabitat and landscape scales (*Microhabitat* and *landscape*). The analyses were performed with the software R.2.8.0 [33].

First, the variables to be included in each model were examined using Spearman's rank correlation (ρ) index to test the correlation between continuous candidate variables. Only non-correlated variables or those with weak correlation (ρ<0.3) were included in the model carried out at each scale. We did not pose a multiple contrast hypothesis and subsequent selection using information criterion (AIC or similar) [34], [35], in order to integrate the results of this study into the meta-analysis presented later in this work. All previous published papers on nesting-habitat selection of the cinereous vulture used the criteria of statistical significance, so we decided to maintain this criterion [13]–[16].

Response variables were binary (nest/random plot) and so we used generalised linear models with binomial family errors and logit-link functions. We looked for overdispersion using the dispersion parameter, which was calculated for each model by dividing the residual deviance by the residual degrees of freedom. Those models showing overdispersion were refitted by quasi-binomial family error [36].

The models were simplified by removing non-significant terms (α = 5%). Once we had determined the statistically significant factors in each model, we subsequently aggregated the non-significant levels of each factor to obtain the “minimal adequate model”, by a stepwise *a posteriori* procedure. If two levels of a factor did not differ significantly and did not improve the fit of the model, they were grouped together [36].

#### Meta-analysis

First, we compared the variability explained by each of the studies, including the present field study results, for evaluating their ability to effectively model habitat selection from a methodological point of view. Hence, a meta-analysis testing the null-hypothesis of no-differences among the mean values of the deviance or variance was performed by using the Cochran's Q statistics of hetereogeneity. Previously, we checked that variance-deviance values of the studies fitted to a Chi-square distribution (χ2 = 136.12; df = 8; p<0.001) [37]. I-squared test quantifies the degree of heterogeneity of the studied values by analysing the percentage of the whole observed differences in the deviance-variance values between studies which are not due to chance [38]. The effect-size of the studied variable (deviance or variance) and its confidence interval at 95% were also evaluated. ANOVA (for categorical variables) and regression (for continuous variables) analyses were performed to assess the influence of sampling procedures on the deviance-variance results. All analyses were performed with the software Comprehensive Meta-Analysis V.2 [20].

On the other hand, we analysed which variables from those studied predicted to be important in habitat selection. Thus, to evaluate the frequency of appearance of each statistically significant variable regarding nest-selection we chose variables that were significant most often by selecting those resulting significant more than once from the whole studies and provided that the variable was significant more than 1/3 of times it was considered. These variables were grouped according to their relationship with *microhabitat*, to the ecological characteristics at landscape level (*landscape*), to climate, to the effect of anthropic activities (*anthropic*) and others.

Subsequently, a Fisher's exact test was used to analyze the proportion of appearance of each type of significant variables (*landscape*, *microhabitat* and *anthropic*) and the characteristics of the studied population (in terms of the *threat level*, *colony size* and *habitat*). The software Statistica 6.1 [39] was used to perform these analyses.

## Results

### Habitat modelling from present field study

In relation to *microhabitat*, the variables *D_tree* and *H_tree* were highly correlated (ρ = 0.58) and we decided to include the first variable in the analysis, together with *Sp_tree* and their first-order interaction (Appendix S2). The levels ‘cork oak’ and ‘juniper’ of the factor *Sp_tree* were not apparently different from each other but were with respect to the level ‘holm oak’. We checked that they could be combined into a single level without any statistically significant variation in the model by analysing changes in null deviance (χ^2^ = −1.29; df = 2; p = 0.041) to derive the minimal adequate model (Table 1). Vultures bred less often in holm oaks than in cork oaks and junipers. The interaction between species and tree diameter was also significant, and indicated that the diameter effect was greater in the case of holm oaks (see Table 1 for further details).

**Table 1:** Dependence of nest-site selection on *microhabitat* characteristics and on variables at *landscape* scale from the selected minimal adequate models.

	Parameter	Effects±SE	z value	p value	Explained deviance
*Microhabitat*	Intercept	−3.7721±1.4289	−2.649	<0.001	0.18
	Sp.(cork oak+juniper) oak+juniper)	3.3882±1.6553	2.047	0.040	
	D_tree	0.1242±0.0397	3.127	<0.001	
	D_tree*Sp. oak+juniper	−0.0905±0.433	−2.088	0.036	

	Parameter	Effects±SE	t value	p value	Explained deviance
*Landscape*	Intercept	2.6212±5.9622	0.440	0.660	0.89
	Slope	0.5549±0.0954	5.813	<0.001	
	Orient_(N+E+W)	−4.0760±0.7230	−5.638	<0.001	
	D_scree	−0.0279±0.0052	−5.290	<0.001	
	Rad25_tree	−0.8642±0.1622	−5.327	<0.001	
	%tree	0.4772±0.08554	5.562	<0.001	
	H_shrub	3.3206±0.8554	3.882	<0.001	
	D_road	0.0018±0.0091	3.500	0.0011	
	D_track	0.0451±0.0090	4.875	<0.001	
	%_shrub	−0.0059±0.0155	−3.854	<0.001	
	Alt	−0.0564±0.0112	−5.014	<0.001	
	%_Qrot	−0.0601±0.0202	−2.971	0.003	
	D_const	0.0065±0.0019	6.043	<0.001	

Effects±SE were calculated considering the reference value of zero for Sp_Tree (olm oak) and the same for the interaction D_tree*Sp; Orient_(S).

At *landscape* scale, we did not include in the model the variables *%_Qsuber* and *%_ othersp*, as they were correlated with *%_Qrot* (ρ = −0.88 and ρ = −0.75, respectively), nor *%_scree-rock* (correlated with *D_scree* ρ = −0.61) nor *Long_tracks* (correlated with *D_track* ρ = −0.75 and *D_road* ρ = 0.36). It was necessary to correct for overdispersion. South-facing slopes were selected for cinereous vulture to locate their nests in advance so northern, eastern and western orientation were joined obtaining a more parsimonious model from the precedent without statistical differences (F = 0.91, df = 2, p = 0.40) in order to simplify the number of levels of this variable. The model, after the simplification, it is shown in Table 1. A greater slope and the closeness of screes had a significant positive effect on nest-site selection; southern facing sites were selected in comparison with other orientations. Greater tree coverage within a radius of 100 m around the studied plot and a higher scrub layer showed a positive effect on nest presence. On the other hand, the presence of trees over 4 m high in a radius of 25 m around the nest as well as a greater cover of holm oak and shrub disfavoured selection by the cinereous vulture. Finally, the nests tended to be far from tracks, roads and human buildings (see Table 1 for further details).

### Meta-analysis from published articles

**Figure 2:**
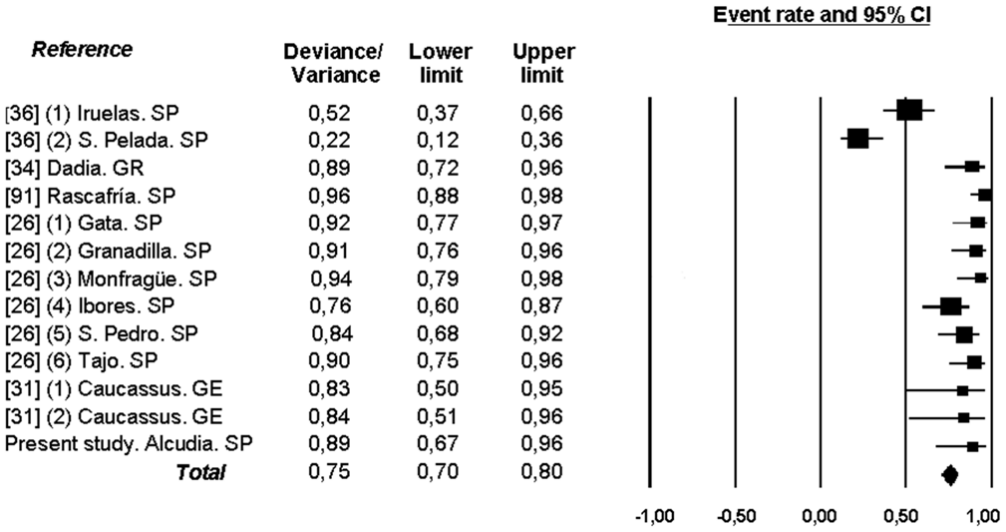
Effect size of the meta-analysis of the deviance-variance values of different studies on the breeding habitat selection of the cinereous vulture *Aegypius monachus*. The studies references, according to Appendix S1, its location (SP = Spain, GR = Greece, GE = Georgia), the values of deviance/variance and their confidence intervals at 95% are presented.

In terms of sampling procedures, significant statistical differences in the deviance/variance existed between colonies (Q = 89.18, df = 12, p<0.001, I^2^ = 86.54, Figure 2). In addition, other factors also influenced the effect size of the deviance/variance reported in each of the analyzed studies: the location of the random plots (Q = 52.81, df = 2, p<0.001, greater deviance/variance when plots are inside the perimeter of breeding colony), whether all data were sampled in a single year (Q = 29.28, df = 1, p<0.001, greater deviance/variance when one-year sampling), the type of random data considered (Q = 47.87, df = 1, p<0.001, greater deviance/variance for points) and the ratio between the number of nests sampled and the number of random samples (Q = 89.18, df = 1, p = 0.002, greater deviance/variance when lesser nest/random ratio).

The variables that were most often selected as statistically significant in nest-site selection for the whole Eurasian studies are presented in Table 2, being a greater slope, bigger diameter of the tree, the presence of scree around the nest-tree, orientation of the slope to south and a longer distance to tracks the five more proportionally and positively related to the presence of cinereous vulture nests. The number and type of significant variables regulating nest-site selection did not relate to the vulnerability of the colony (Table 3). Colony size conditioned the number of significant variables in relation to landscape such that in the largest colonies these variables were of greater importance (Table 3). In terms of habitat type, human activities had a greater negative effect in colonies in Mediterranean forests of *Quercus* sp. than in colonies located in pines (Table 3).

**Table 2:** Most frequent significant variables resulting from nest-site selection in 16 cinereous vulture breeding colonies.

Variable	Type	Relation	n_signif_	n_stud_	Proportion
*Slope*	Landscape	+	8	12	0.67
*Tree diameter*	Microhabitat	+	4	6	0.67
*Scree presence*	Landscape	+	2	3	0.67
*Orientation south*	Landscape	+	3	5	0.60
*Distance to track*	Anthropic	+	7	12	0.58
*Cork oak cover*	Landscape	+	3	6	0.50
*Human population index*	Anthropic	−	3	6	0.50
*Holm oak cover*	Landscape	−	3	6	0.50
*Position on upper third of the slope*	Landscape	+	2	4	0.50
*Tree height*	Microhabitat	+	3	7	0.43
*Distance to road*	Anthropic	+	5	12	0.42
*Orientation east*	Landscape	+	3	5	0.60
*Distance to nearest nest*	Others	+	2	5	0.40
*Altitude*	Landscape	+	6	16	0.37

*Relation* indicates the type of relationship (positive or negative) of the variable with the selection by the cinereous vulture; (n _signif_) shows the number of colonies for which the variables was statistically significant; (n_stud_) indicates the number of colonies in which the variable was studied, and *Proportion* shows the ratio of the two previous numbers (n_1_/n_2_). The table includes variables that were statistically significant more than once and in more than one third of the studied colonies.

**Table 3:** P-values of the Fischer's exact test of the relation between variables that were significant in higher proportion in the different studies of nest-site selection of cinereous vulture (grouped in variables related to *microhabitat*, to *landscape* scale and to human interactions- *anthropic*-) to the threat level of the studied breeding colonies, to their colony size and to their type of habitat.

	Type of significant variable of the nesting-habitat selection		
Characteristics of the studied breeding colonies	*Microhabitat*	*Landscape*	*Anthropic*
*Threat level*	0.993	0.693	0.941
*Colony size*	0.561	0.015	0.552
*Habitat*	0.275	0.314	0.077

## Discussion

### Habitat selection

The present field study and the meta-analysis showed that the cinereous vulture selected nest-sites in large trees, on steep, south-facing slopes, close to screes and away from human infrastructures or anthropogenic factors than can provoke disturbances. Steeper slopes determine nest-site selection in the cinereous vulture since disturbances are less likely to occur in rugged areas [40], [41]. The largest trees cope better with the weight of nests and are easier to land on and take off from [16], [17]. This robustness and height, often found around screes [42] and in old cork oaks, as well as the positively selected situation on the upper third of a hillside, ease the detection of predators and other sources of disturbance [12], [43].

South- and east-facing slopes are selected as nest sites, probably because there are higher trees and better climate at a local scale [44]. Nonetheless, no climate-related variables were relevant in the general nest-site choice in the cinereous vulture, either due to the inter-annual heterogeneity of this feature, the fact that at local scale these variables do not predict adequately variations in ecological processes [45] or because not all the same variables were examined in the studies that we analyzed. Anthropic factors are very important in habitat selection, as occurs in other species that are wary of humans [46], [47].

Nevertheless, the impact of human activities is a complex issue that should be case-by-case evaluated in relation to each breeding colony, since the secular management practices, the degree of habituation to human presence, the social awareness to this potential beneficial species or the ecological and biogeographical conditions may nuance its real influence [48], [49]. In this sense, both vegetation and the availability and characteristics of trophic resources, as well as the impact of human activities, vary between regions approximately along a geographical gradient [50], [51], although this species does have a certain plasticity in its ecological requirements [52]. These differences can be very marked as is shown by the fact that this species breeds on cliffs in Mongolia and Russia [53], thereby demonstrating that the factors affecting life histories in a single species with a wide range can be very heterogeneous [54], [55].

### Modelling and methodological conclusions

First, the number of studies analyzed (n = 13) was small to be able to draw definitive conclusions so patterns regarding the most efficient sampling approaches (see below) might be considered in relation to this low sample size. This is a common gap for meta-analysis works whose analytical procedures allow to integrate the onset of low sample sizes [32], [37]. Nevertheless, this type of analysis of a single species has never previously been conducted in such a wide geographical context [56].

The total number of variables under study influences the results of the models and the variability that they reflect [57], [58]. This implies that in habitat-modelling studies both the number and type of variables must be carefully chosen beforehand [59], [60]. In this sense, it is worth highlighting the fact that it is important to choose the most explanatory variables and/or those that are easily repeatable [4]; otherwise, there is a risk that the processes will not provide information regarding the proposed objectives. This deficiency is especially relevant in the study of climate, since many different variables are used to study the same factors [e.g. temperature, humidity, rainfall, frost and wind) and provide only scattered and inconclusive information [30], [61]. On the other hand, the influence of other factors such as the effects of global change, the use of integrating variables in ecological processes, diseases and certain biochemical factors [62], [63] could modify the tendencies of the results obtained and could lead to the application of better planned and more efficient conservation policies [64]. Our study followed the patterns of choice of variables commonly considered in previous works, both in number and type, so it was not possible to include more interesting and complex analysis in the field work, which may reveal other significant factors.

The location of the random sampling points influences the variability detected in the study. Thus, if the random plots are situated within the colony the information obtained will be more detailed in terms of factors operating at a local scale. On the other hand, if random points are chosen at a scale that includes heterogeneous types of habitat, some of the more general variables are more likely to be significant [e.g. altitude], [ slope or vegetation cover]; [ 13,57,65]. In our case we selected *a priori* the inner perimeter of the studied breeding colony as the framework for analysing differences between random and nests plots. One main objective was to assess differences in the selected habitat characteristics at precise level and thus, to show the sensitive factors for the breeding of cinereous vulture and to recommend the most suitable locations for developing land-use practices to local managers.

Our results reveal that if data are gathered during just one breeding season, the variability explained by the models increases, possibly because uncontrolled aspects such as inter-annual change regulating nest-site choice, weather conditions and individual behaviour are avoided [66]. Nevertheless, other studies have shown that bias could be reduced and variability in the results increased when long-term data incorporating temporary dynamics are analyzed [58], [67]. Bias-variance trade-off determines general model fitting [58], [66] and it is not possible to know exactly the bias integrated in each of the meta-analysed studies. Thus, our results should be interpreted with caution in this regard.

The election of points as random plots allows us to reflect greater deviance/variance in the model than if polygons are employed. Thus, results can throw light more accurately on questions regarding nest selection by species [16], [68].

Lastly, it is important that the proportion between the sampled nests and the random plots is as balanced as possible, although if there is an unbalance it should be in favour of the random plots. In this way, when the relationship sample/random approaches 0, the explained variability increases [57], [66], [69]. Therefore, the election of a lesser number of random plots in relation to the nests in our field study could reduce the explained deviance (Table 1).

### Implications for conservation

The results obtained show that habitat type and the size of breeding colony affect the type of variables that most influences nest-site selection. Thus, human activities have greater incidence in colonies situated in Mediterranean oak forests. This may be due to the relative ease of access to the colony, to the habitat quality or to the existence of the additional conservation problems [70], [71]. In addition, it is possible that cinereous vultures may have a closer relationship with human activities in specific habitats such as pine forests, which have been exploited for a long time in a sustainable way with respect to the requirements of species present [72], [73]. In Mediterranean oak forests, on the other hand, few human economic activities are undertaken during the breeding season (except cork harvesting) and so it is possible that in these environments the species is more sensitive to human presence [43].

Despite the existence of various forest management models [73], the results of our meta-analysis suggest the need to implement different generalized management policies in temperate forests of the Palaearctic: 1) Mature forests must be given priority in forest protection as they act as source of resources and diversity [48], [72], [74]. Our results showed that areas with greater trees and tree cover are the most valuable type of forests for the breeding cinereous vultures. 2) Economic activities often determine habitat selection by threatened species [75] and so exclusion areas should be established for the most threatening activities and/or agreements should be reached to make human activities compatible [12]. Cinereous vultures tend to locate their nests as far as possible from human presence so one of the management priorities should be the regulation of such activities [71]. 3) It is advisable to coordinate and to standardize the data sampling procedures in advance when planning habitat modelling studies for the same species at different geographical scales. It is thus interesting to make the effort of developing scientific and technical working groups integrated by experts and researchers dealing with species of conservation concern [6], [74]. 4) The analysis of ecological processes that include variables that have not been taken into account to date in habitat modelling like those related to climate change, parasites-diseases or biochemical properties must be encouraged [64] and scientific evidence-based criteria must be applied on the basis of these specific studies [76].

According to these conclusions, the knowledge of habitat selection in indicator-endangered species is very valuable for optimizing evidence-based conservation actions [56], [76], [77]. Specifically, the modelling of species requirements should be undertaken for both conservation actions *ex situ* and *in situ*. Species reintroduction programmes should take into account the analysis of global patterns of habitat selection [78], [79] and so studies evaluating ecological requirements are of great relevance for carrying out population viability analyses [80], [81]. In the case of the cinereous vulture, it could be even more important given that one of the main conservation objectives for this species is the establishment of biological corridors that will connect currently isolated Palearctic populations [21], [82] through reintroduction projects (e.g., in the Pyrenees, France, Balkan Peninsula).

## Supporting Information

Appendix S1 Information contained in the articles consulted for the meta-analysis of the variation in the nest-site selection in the cinereous vulture *Aegypius monachus*. The variables considered for conducting the different analysis are shown (see *methods* for further information).
(DOC)

Appendix S2 Models evaluated for the analysis of nest-site selection by the cinereous vulture *Aegypius monachus* in the colony in Alcudia Natural Park, Spain. The scale of analysis, categorical (*factors included*) and continuous variables, the pairs of variables that were found to be correlated using the Spearman test (ρ), the interactions between pairs of variables included in the models, and the number of generalized linear models resulting that were analyzed are shown. Each model was built including all factors, non-correlated continuous variables and those with interactions with ρ<0.30, and only one of correlated variables each model.
(DOC)
