# Supplementary material for: Effect of Methodological and Ecological Approaches on Heterogeneity of Nest-Site Selection of a Long-Lived Vulture
Source: PLoS One. 2012 Mar 8;7(3):e33469. doi: 10.1371/journal.pone.0033469 (PMC3297632; doi:10.1371/journal.pone.0033469)
Supplement: Appendix S1 — Information contained in the articles consulted for the meta-analysis of the variation in the nest-site selection in the cinereous vulture Aegypius monachus. The variables considered for conducting the different analysis are shown (see methods for further information). (DOC) [file pone.0033469.s001.doc]

| **Reference** | **Breeding colony** | **Number pairs (colony size code)** | **Threat level** | **Habitat** | **Sample** | **Sampling in same year** | **Nest samples** | **Random samples** | **Nest samples/ random samples** | **Location random plots** |
| --- | --- | --- | --- | --- | --- | --- | --- | --- | --- | --- |
| [17] | Sª Guadarrama I (Spain) | 11 (1) | 2 | Pine | Point | Yes | 16 | 31 | 0,52 | 2 |
| [17] | Sª Guadarrama II (Spain) | 7 (1) | 2 | Pine | Point | Yes | 13 | 37 | 0,35 | 2 |
| [16] | Iruelas (Spain) | 84 (2) | 3 | Pine | Polygon | No | 47 | 23 | 2,04 | 2 |
| [16] | Sª Pelada (Spain) | 96 (2) | 3 | Pine | Polygon | No | 104 | 104 | 1,00 | 2 |
| [18] | Cabañeros (Spain) | 165 (3) | 3 | Oak | Point | No | 486 | 486 | 1,00 | 1 |
| [15] | Dadia (Greece) | 30 (1) | 1 | Pine | Point | No | 25 | 25 | 1,00 | 2 |
| [19] | Rascafría (Spain) | 74 (2) | 3 | Pine | Polygon | No | 153 | 153 | 1,00 | 3 |
| [13] | Gata-Hurdes (Spain) | 81(2) | 3 | Pine | Point | Yes | 42 | 562 | 0,07 | 3 |
| [13] | Granadilla (Spain) | 18 (1) | 2 | Pine | Point | Yes | 19 | 562 | 0,03 | 3 |
| [13] | Monfragüe (Spain) | 287 (3) | 3 | Oak | Point | Yes | 169 | 562 | 0,30 | 3 |
| [13] | Ibores (Spain) | 52 (2) | 2 | Oak | Point | Yes | 30 | 562 | 0,05 | 3 |
| [13] | San Pedro (Spain) | 336 (3) | 3 | Oak | Point | Yes | 249 | 562 | 0,44 | 3 |
| [13] | Tajo Internacional (Spain) | 63 (2) | 3 | Oak | Point | Yes | 42 | 562 | 0,07 | 3 |
| [14] (1) | Caucassus (Georgia) | 30* (1) | 1 | Pine | Point | No | 31 | 70 | 0,44 | 3 |
| [14] (2) | Caucassus (Georgia) | 30* (1) | 1 | Pine | Point | No | 12 | 100 | 0,12 | 1 |
| Present study | Umbría Alcudia (Spain) | 129 (3) | 3 | Oak | Point | Yes | 155 | 85 | 1,82 | 1 |
|  |  |  |  |  |  |  |  |  |  |  |
|  |  | **Studied variables** | | | | |  |  | | |
| **Reference** | **Breeding colony** | **Climatic** | **Nest-site level** | **Landscape** | **Antropic** | **Population** | **Total studied variables** | **Significant variables** | **Significant/ studied variables** | **Deviance or variance** |
| [17] | Sª Guadarrama I (Spain) | 0 | 15 | 3 | 2 | 1 | 21 | 13 | 0,65 | - |
| [17] | Sª Guadarrama II (Spain) | 0 | 15 | 3 | 2 | 1 | 21 | 5 | 0,25 | - |
| [16] | Iruelas (Spain) | 0 | 0 | 22 | 20 | 0 | 42 | 3 | 0,07 | 0,52 |
| [16] | Sª Pelada (Spain) | 0 | 0 | 22 | 20 | 0 | 42 | 3 | 0,07 | 0,22 |
| [18] | Cabañeros (Spain) | 0 | 0 | 4 | 2 | 0 | 6 | 5 | 0,83 | - |
| [15] | Dadia (Greece) | 0 | 12 | 14 | 5 | 0 | 31 | 10 | 0,32 | 0,89 |
| [19] | Rascafría (Spain) | 6 | 2 | 59 | 5 | 1 | 73 | 33 | 0,45 | 0,96 |
| [13] | Gata-Hurdes (Spain) | 19 | 0 | 11 | 6 | 0 | 36 | 5 | 0,14 | 0,92 |
| [13] | Granadilla (Spain) | 19 | 0 | 11 | 6 | 0 | 36 | 5 | 0,14 | 0,91 |
| [13] | Monfragüe (Spain) | 19 | 0 | 11 | 6 | 0 | 36 | 6 | 0,17 | 0,94 |
| [13] | Ibores (Spain) | 19 | 0 | 11 | 6 | 0 | 36 | 6 | 0,17 | 0,76 |
| [13] | San Pedro (Spain) | 19 | 0 | 11 | 6 | 0 | 36 | 13 | 0,36 | 0,84 |
| [13] | Tajo Internacional (Spain) | 19 | 0 | 11 | 6 | 0 | 36 | 6 | 0,17 | 0,90 |
| [14] (1) | Caucassus (Georgia) | 1 | 2 | 5 | 3 | 0 | 11 | 2 | 0,18 | 0,83 |
| [14] (2) | Caucassus (Georgia) | 1 | 2 | 5 | 3 | 0 | 11 | 3 | 0,27 | 0,84 |
| Present study | Umbría Alcudia (Spain) | 0 | 3 | 14 | 4 | 0 | 21 | 15 | 0,71 | 0,89 |
