# Supplementary material for: Effect of Methodological and Ecological Approaches on Heterogeneity of Nest-Site Selection of a Long-Lived Vulture
Source: PLoS One. 2012 Mar 8;7(3):e33469. doi: 10.1371/journal.pone.0033469 (PMC3297632; doi:10.1371/journal.pone.0033469)
Supplement: Appendix S2 — Models evaluated for the analysis of nest-site selection by the cinereous vulture Aegypius monachus in the colony in Alcudia Natural Park, Spain. The scale of analysis, categorical (factors included) and continuous variables, the pairs of variables that were found to be correlated using the Spearman test (ρ), the interactions between pairs of variables included in the models, and the number of generalized linear models resulting that were analyzed are shown. Each model was built including all factors, non-correlated continuous variables and those with interactions with ρ<0.30, and only one of correlated variables each model. (DOC) [file pone.0033469.s002.doc]

| **Scale of analysis** | **Factors included** | **Continuous variables included** | **Pairs of cont. variables**  **with ρ>0.30** | **Interactions included** |
| --- | --- | --- | --- | --- |
| *Microhabitat* | Sp_tree | H_tree; D_tree | H_tree & D_tree | Sp_tree*D_tree |
| *Landscape* | Scree; Orient | Alt; Slope; D_scree; Rad25_tree; H_shrub; %tree; %shrub; %past; %scree-rock; %Qsuber; %Qrot; %other; long_track; D_track; D_road; D_const | %scree-rock & D_scree  %Qrot & %Qsuber  %Qrot & %othersp  long_track & D_track  Long_track & D_road | %shrub*H_shrub  D_road*D_track |
